# A Neurophysicist's view to the evolving brain based on neural physiological measurements; diagnosis, development, and accelerated cognitive processing

**DOI:** 10.3389/fncom.2013.00092

**Published:** 2013-07-05

**Authors:** Cartik Sharma

**Affiliations:** Carpen ConsultingToronto, ON, Canada

We present a novel platform to facilitate the growth of applied quantum information processing for neural circuitry, neural stimulation, and cognitive engineering. Cognitive computation has historically been studied by rich metaphors of language constructs, computational models for information processing, and semantic interpretations. Cortical interactions at the cellular and sub cellular level have been established for various cognitive tasks by behavioral and computational neuroscientists. This has enabled us to explore cognitive circuitry given a mathematical and psychophysical basis for information processing and neural decision making.

We invite original works in applied physics, computing, and information theory pertaining to neural choice specifically with the intent of developing non-linear mathematical models and theory for neural computation.

Various modalities have emerged for physiological processing of neural signals with the premise of measuring electrical or magnetic activity for cortical source localization for simple cognitive tasks. Primary amongst these are EEG and MEG that measure electrical current and magnetic field strengths relative to ambient environments of signal to noise ratios.

We propose to extend the physiological measurement paradigm toward creating computational and physics-based building blocks for neural information processing and cognitive function. The goals are dual, to apply our learning of neural measurements and observations toward a constructional cortical framework and improve on current neural processing model for cognitive enhancement, rehabilitation, and cognitive evolution. Efforts are orchestrated toward mathematically sound arguments and quantum information theoretic frameworks to explain neural information processing and cognitive circuitry. Theories such as the Hebbian rule for learning, “Cells that wire together fire together,” are famous for their considerations in foundational cognitive neuroscience and address simplistic rules for cognitive behavior.

We intend to create computational models for learning and neurodevelopment toward explaining complex cognitive decisions and improving cognitive function with emerging brain computing interfaces. We invite quantum simulation models for neural computing toward strides in computational neuroscience. Special preference would be given to strong foundations in mathematical neuroscience for objectifying cognitive function as relates to human brain.

